# Myoprotective effects of bFGF on skeletal muscle injury in pressure-related deep tissue injury in rats

**DOI:** 10.1186/s41038-016-0051-y

**Published:** 2016-08-17

**Authors:** Hongxue Shi, Haohuang Xie, Yan Zhao, Cai Lin, Feifei Cui, Yingying Pan, Xiaohui Wang, Jingjing Zhu, Pingtao Cai, Hongyu Zhang, Xiaobing Fu, Jian Xiao, Liping Jiang

**Affiliations:** 1 School of Pharmaceutical Sciences, Key Laboratory of Biotechnology and Pharmaceutical Engineering, Wenzhou Medical University, Wenzhou, 325035 People’s Republic of China; 2 Department of Nursing School, Wenzhou Medical University, Wenzhou, 325035 People’s Republic of China; 3 Department of Nursing, The Affiliated Xinhua Hospital of Shanghai Jiaotong University School of Medicine, Shanghai, 200092 People’s Republic of China; 4 Department of Burns, The First Affiliated Hospital, Wenzhou Medical University, Wenzhou, 325035 People’s Republic of China; 5 Department of Nursing, The Affiliated Dongyang People’s Hospital of Wenzhou Medical University, Jinhua, 322100 People’s Republic of China; 6 Wound Healing and Cell Biology Laboratory, Institute of Basic Medical Science, Trauma Center of Postgraduate Medical School, Chinese PLA General Hospital, Beijing, 100853 People’s Republic of China

**Keywords:** Pressure ulcer, Skeletal muscle injury, bFGF, Regeneration, PI3K/Akt/mTOR

## Abstract

**Background:**

Pressure ulcers (PUs) are a major clinical problem that constitutes a tremendous economic burden on healthcare systems. Deep tissue injury (DTI) is a unique serious type of pressure ulcer that arises in skeletal muscle tissue. DTI arises in part because skeletal muscle tissues are more susceptible than skin to external compression. Unfortunately, few effective therapies are currently available for muscle injury. Basic fibroblast growth factor (bFGF), a potent mitogen and survival factor for various cells, plays a crucial role in the regulation of muscle development and homeostasis. The main purpose of this study was to test whether local administration of bFGF could accelerate muscle regeneration in a rat DTI model.

**Methods:**

Male Sprague Dawley (SD) rats (age 12 weeks) were individually housed in plastic cages and a DTI PU model was induced according to methods described before. Animals were randomly divided into three groups: a normal group, a PU group treated with saline, and a PU group treated with bFGF (10 μg/0.1 ml) subcutaneously near the wound.

**Results:**

We found that application of bFGF accelerated the rate of wound closure and promoted cell proliferation and tissue angiogenesis. In addition, compared to saline administration, bFGF treatment prevented collagen deposition, a measure of fibrosis, and up-regulated the myogenic marker proteins MyHC and myogenin, suggesting bFGF promoted injured muscle regeneration. Moreover, bFGF treatment increased levels of myogenesis-related proteins p-Akt and p-mTOR.

**Conclusions:**

Our findings show that bFGF accelerated injured skeletal muscle regeneration through activation of the PI3K/Akt/mTOR signaling pathway and suggest that administration of bFGF is a potential therapeutic strategy for the treatment of skeletal muscle injury in PUs.

**Electronic supplementary material:**

The online version of this article (doi:10.1186/s41038-016-0051-y) contains supplementary material, which is available to authorized users.

## Background

Pressure ulcers (PUs) are defined as localized breakdown ulcerated tissue caused by sustained mechanical pressure in the body support interface. The prevalence of PUs in the USA is 3 million, and PUs are a particularly common problem among older adults in all health care settings [1]. PUs are a major source of morbidity, mortality, and health care costs. The annual cost of PUs in the USA is estimated to be between USD 9.1 billion and USD 11.6 billion [2] and is expected to increase with the drastic growth of the elderly population, the cohort who are most susceptible to PUs.

The term of “Deep Tissue Injury (DTI)” was put forward by the National Pressure Ulcer Advisory Panel (NPUAP) to define a unique type of PUs that develop as a consequence of damage to underlying soft tissues, such as muscles and bones. DTI develop into cavity-shaped large open wounds that when undiagnosed or not treated in a timely manner can lead to complications such as sepsis, myocardial infarction, renal failure, and multiple organ dysfunction [3]. Pressure, shear, and ischemia have been identified as causes of PUs, and at the cellular and molecular levels, oxidative stress, autophagy, and apoptosis play important roles in the development and progression of pressure-induced DTI [4, 5].

Skeletal muscle injury and repair are complex processes, involving degeneration, inflammation, regeneration, and fibrosis. Skeletal muscle tissue regeneration occurs through the activation of satellite cells, a population of quiescent myogenic cells, located between the basal lamina and plasma membrane of the muscle fiber [6, 7]. Upon skeletal muscle injury, satellite cells proliferate and differentiate into mature myotubes that facilitate skeletal muscle regeneration [8]. However, it is generally assumed that the mere presence of satellite cells is insufficient to ensure rapid functional recovery of injured muscle. The release of appropriate growth factors, cytokines, and the establishment of a suitable microenvironment are also important in an effective myogenic response [9, 10].

Among the molecules thought to be involved in the myogenic response, fibroblast growth factors (FGFs) have diverse roles in cell proliferation, differentiation, migration, and survival in most mesoderm and neuroectoderm-derived cells [11]. The FGF family protein basic fibroblast growth factor (bFGF) is a potent mitogen and performs different biological roles in tissue repair and regeneration [12, 13]. A study has shown that while bFGF messenger RNA (mRNA) transcripts can be detected in myotubes in non-injured muscle, in a muscle injury model, bFGF mRNA can be observed not only in myoblasts but also in degenerating and regenerated myotubes, suggesting that bFGF plays a role in the myogenic program [14]. Though bFGF is widely used to treat diabetic ulcers, gastric ulcers, surgical wounds, burns, and even spinal cord injury [15], few studies have focused on the role of bFGF in DTI. Thus, we aimed to determine whether bFGF could ameliorate skeletal muscle injury and improve regeneration of injured skeletal muscle.

Our previous study showed that bFGF promotes full-thickness excisional wound healing and reduces scar formation [16]. In the present study, application of bFGF was extended to ischemia/reperfusion-mediated DTI PU in rats. The effects of bFGF treatment on DTI were evaluated by measuring cell proliferation, angiogenesis, myogenesis, collagen deposition, and activation of signaling pathways.

## Methods

### Reagents and antibodies

Anti-Akt, anti-p-Akt (Ser473), anti-CD31, anti-actin primary antibodies, and appropriate secondary antibodies were purchased from Santa Cruz Biotechnology (Santa Cruz, CA, USA). Anti-mTOR and anti-p-mTOR antibodies were purchased from Cell Signaling Technology (Danvers, MA, USA). Anti-myogenin and anti-MyHC antibodies were obtained from Abcam (Abcam, Cambridge, MA). All other reagents used were obtained from Sigma-Aldrich (St. Louis, MO, USA).

### Animal treatment

This study was reviewed and approved by the Ethics Committee for Experimental Animals of Wenzhou Medical University. Male Sprague Dawley (SD) rats (age 12 weeks) were purchased from the Animal Center of the Chinese Academy of Sciences and housed under standard conditions. Animals were individually housed in plastic cages, and a DTI PU model was induced according to methods described in a previous study [17]. Additional file 1 provided detailed information on DTI model and experiment design. Animals were randomly divided into three groups: a normal group, a PU group treated with saline, and a PU group treated with bFGF. For the PU group treated with bFGF, bFGF solution (10 μg/0.1 ml) was injected subcutaneously near the wound on the left side every other day beginning at 0 day after DTI model, while equivalent volumes of saline were injected on the right side. The reason for choosing high dose of bFGF due to our pilot study demonstrated that 1.0 and 5.0 μg dose of bFGF could not promote deep tissue wound healing after 14-day treatment. Photos of the experimental wounds were taken with a ruler at the different time points, and wound area was assessed using NIH Image J software.

### Hematoxylin–eosin staining and Masson’s trichrome staining

Histopathological examination by hematoxylin–eosin (HE) staining and interstitial collagen deposition examination by Masson’s trichrome staining were performed on formalin-fixed, paraffin-embedded tissue as previously described [18]. Images were acquired at ×200 magnification on a Nikon digital camera.

### Immunohistochemistry

Immunohistochemical analyses were performed with the use of primary antibodies against CD31 (1:300) and PCNA (1:200) on formalin-fixed, paraffin-embedded tissue as previously described [18]. Images were acquired at ×200 magnification on a Nikon digital camera.

### Immunofluorescence

Immunofluorescence analyses were performed with the use of primary antibodies against CD31 (1:100) or myogenin (1:250) on formalin-fixed, paraffin-embedded tissue as previously described [18]. Cellular nuclei were counterstained with Hoechst 33258. Labeled sections were imaged at ×200 magnification using a Nikon digital camera.

### Western blot analysis

Total protein was extracted from skeletal muscle using protein extraction reagents. Western blot analyses were performed with the use of primary antibodies against myogenin (1:500), MyHC (1:500), Akt, p-Akt (1:500), mTOR, p-mTOR (1:1000), or actin (1:1000) as previously described [18]. The protein bands were visualized and analyzed using the ChemiDicTM XRS+ Imaging System (Bio-Rad Laboratories, Hercules, CA, USA).

### Statistical analysis

Data are presented as mean ± SEM. Statistical significance was determined by two-way analysis of variance (ANOVA) test for comparison of three or more experimental conditions. For all statistical comparisons, *p* values of less than 0.05 were considered statistically significant.

## Results

### Administration of bFGF decreased wound area

We found that in rats with experimentally induced DTI PUs, local subcutaneous injection of bFGF significantly decreased wound areas starting at the seventh day after DTI induction. The reduction in wound area was consistently observed until the end of the observation period, which was the 21st day after DTI (Additional file 1: Fig. S2A). Initial wound size was consistent between groups, as wound area of saline was 72.97 ± 1.181 mm^2^ and that of bFGF-treated rats was 73.11 ± 1.275 mm^2^ (*P* > 0.05) on the day of wound induction. For the saline and bFGF groups’ wound areas on the 4th, 7th, 14th, and 21st days after DTI induction were as follows: 4th day, 50.60 ± 3.316 mm^2^ vs. 44.46 ± 2.197 mm^2^ (*P* < 0.05); 7th day, 47.50 ± 1.467 mm^2^ vs. 37.77 ± 1.110 mm^2^ (*P* < 0.05); 14th day, 33.73 ± 1.326 mm^2^ vs. 16.64 ± 1.197 mm^2^ (*P* < 0.05); and 21st day, 22.89 ± 0.835 mm^2^ vs. 10.32 ± 0.447 mm^2^ (*P* < 0.05), respectively (Additional file 1: Fig. S2B).

### bFGF improved muscle regeneration and inhibited fibrosis

**Fig. 1:**
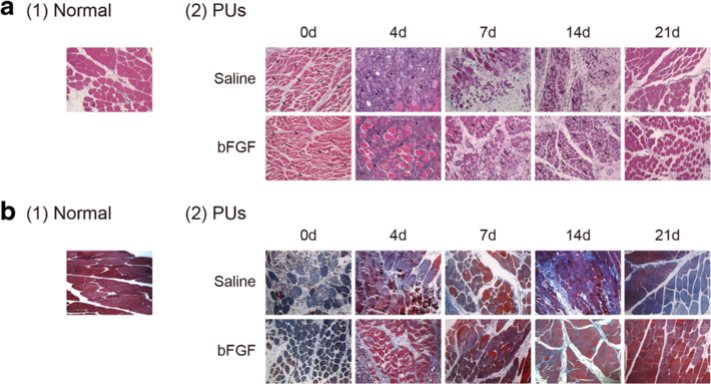
bFGF improved muscle regeneration and inhibited fibrosis. **a** HE staining. The elevation of area of interstitial space (*star*), structural fractured (*arrow*), and mild edema were found in injured muscle tissues on the 0th day. Numerous of inflammatory cells infiltrating (*triangle*) were found in saline group, while bFGF inhibited inflammatory cells infiltrating (*triangle*) on the 4th day. bFGF increased centro-nucleated myofibers (*arrow*) on the 7th and 14th days. **b** Observation of fibrosis distributed in injured muscle tissue by Masson’s trichrome staining. Injured muscle tissues containing collagen deposition (*blue*) and myofibers (*red*) were analyzed. Injured muscle tissues treated with bFGF showed a significant reduction in collagen deposition and increased myofibers within the areas of injury compared with saline group on the 7th, 14th, and 21st days

HE staining was carried out to examine tissue histology at various time points after DTI induction. Broadened interstitial space, structural fractures, and even mild edema were observed in injured muscle tissue compared to normal uninjured tissue, suggesting the degeneration of skeletal muscle in regions of compressed muscle in PUs (Fig. 1a). In addition, while high infiltration of inflammatory cells likely intermingled with proliferating myoblasts and other cell types (e.g., pericytes and fibro-adipogenic precursors) was observed in injured rat administrated saline, the degree of inflammatory cell (mixed population of neutrophils and macrophages) infiltration was reduced on the 4th day after injured in rats treated with bFGF. Few newly regenerated myotubes and myofibers (centro-nucleated), which are the index of muscle regeneration, were found in saline group, while application of bFGF obviously increased the centro-nucleated myofibers on the 7th and 14th days after injury. In addition, in the bFGF group, most damaged myofibers were cleared and replaced by newly formed myofibers containing centralized nuclei as compared to saline group. This was evidenced by a smaller number of interstitial nuclei on the 14th day after DTI. Furthermore, interstitial space was smaller, and skeletal muscle cells were more uniform on the 21st day after injury in the bFGF-treated rats compared to the saline-treated rats.

To characterize the fibrotic area in injured muscle tissues, Masson’s trichrome staining was performed. In the bFGF group, collagen deposition, a measure of fibrosis, was notably lower than that in the saline group on the 7th, 14th, and 21st days after injury, while no difference in collagen deposition was observed on the 4th day, but more myofibers were found in bFGF-treated group (Fig. 1b). These results indicate that the administration of bFGF enhanced injured skeletal muscle regeneration, prevented collagen deposition, and improved muscle recovery after injury.

### Enhancement of proliferation and angiogenesis by bFGF administration

**Fig. 2:**
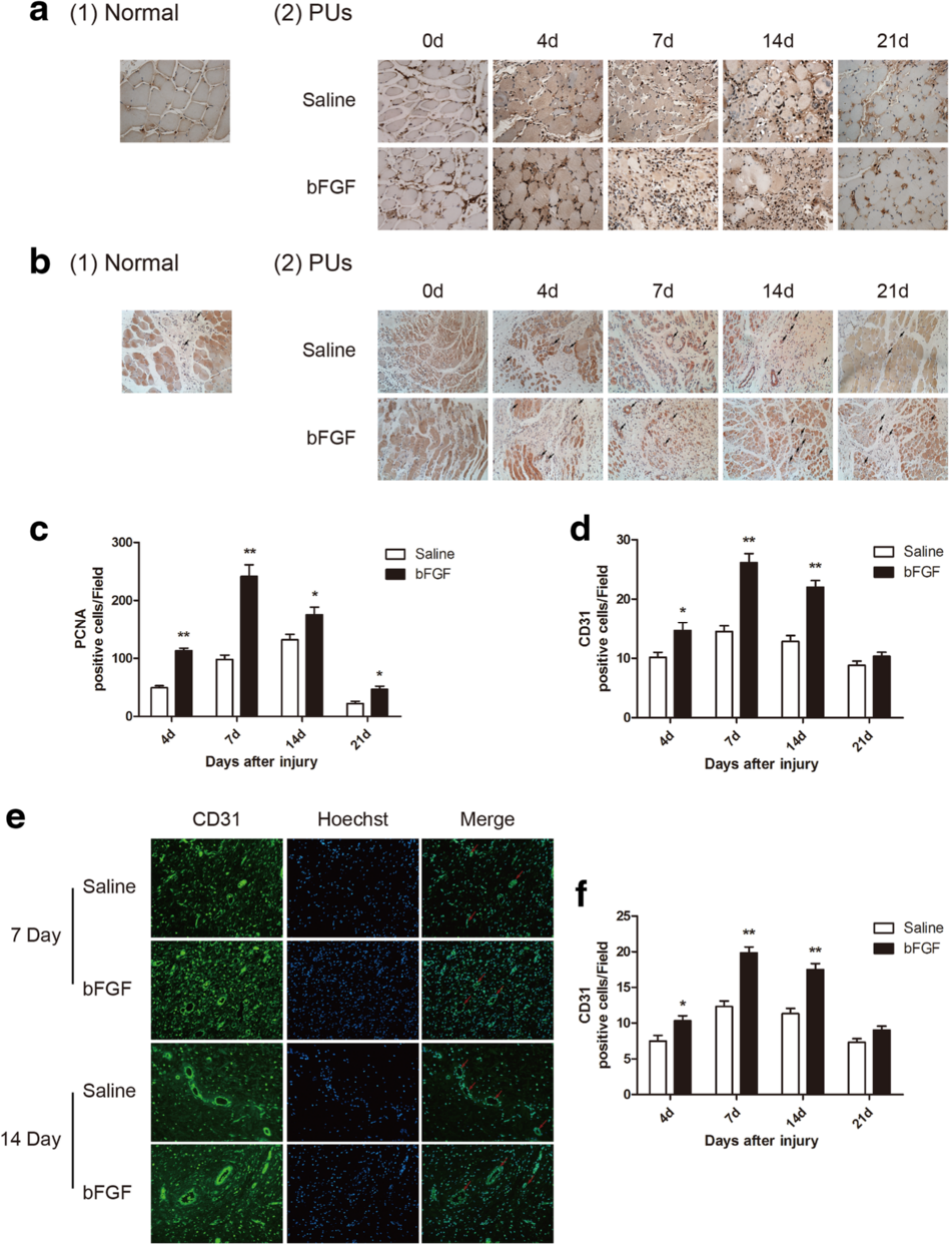
Enhancement of proliferation and angiogenesis by bFGF administration. **a** Photographs showing PCNA immunohistochemistry in saline group and bFGF group (×200). **b** Photographs showing CD31 immunohistochemistry (*arrow*) in two groups (×200). **c**, **d** Graph of PCNA and CD31 immunohistochemistry in two groups. **e** Photographs showing CD31 immunofluorescence (*arrow*) on the 7th and 14th days in two groups (×200). **f** Graph of CD31 immunofluorescence in two groups. Data are expressed as mean ± SEM (*n* = 6). *P < 0.05 compared with saline group

Our immunohistochemistry results showed that the cell proliferation marker proliferating cell nuclear antigen (PCNA)-positive cells were found both in normal group and PU group, the number of PCNA-positive cells was much higher after DTI, suggesting compensatory repair was activated after injury. The number of PCNA-positive cells was increased 2.3-fold on the 4th day after injury (*P* < 0.01), peaked on the 7th day (2.5-fold, *P* < 0.01), and was induced 1.3-fold (*P* < 0.05), and 2.1-fold (*P* < 0.05) on the 14th and 21st days after bFGF treatment, respectively. These results suggest that bFGF contributes to myocyte proliferation and regeneration in damaged skeletal muscle tissue (Fig. 2a, c). Similar temporal change expression was observed for CD31, an endothelial cell marker. The number of CD31-positive cells was up-regulated 1.4-fold (*P* < 0.05) on the 4th day, peaked on the 7th day (1.8-fold, *P* < 0.05), and was induced 1.7-fold (*P* < 0.05) and 1.2-fold (*P* > 0.05) on the 14th and 21st days after the administration of bFGF, respectively (Fig. 2b, d). Capillary density was detected by CD31 immunofluorescence staining. Consistent with previous data, labeling of vasculature by CD31 immunofluorescence showed that bFGF treatment induced greater neovascularization around injury area compared to saline administration. Treatment with bFGF induced a 1.4-fold (*P* < 0.05), 1.6-fold (*P* < 0.05), 1.5-fold (*P* < 0.05), and 1.2-fold (*P* > 0.05) increase in the number of vascular at 4th, 7th, 14th, and 21st days after injury, respectively, suggesting that bFGF effectively promoted angiogenesis and improved the conditions of ischemia reperfusion injury in PUs (Fig. 2e, f). Taken together, the results demonstrated that the level of neovascularization was much higher in bFGF group on the 7th and 14th days after injury, likely due to the known role of bFGF in facilitating angiogenesis.

### bFGF increased expression of myogenic markers myogenin and MyHC

**Fig. 3:**
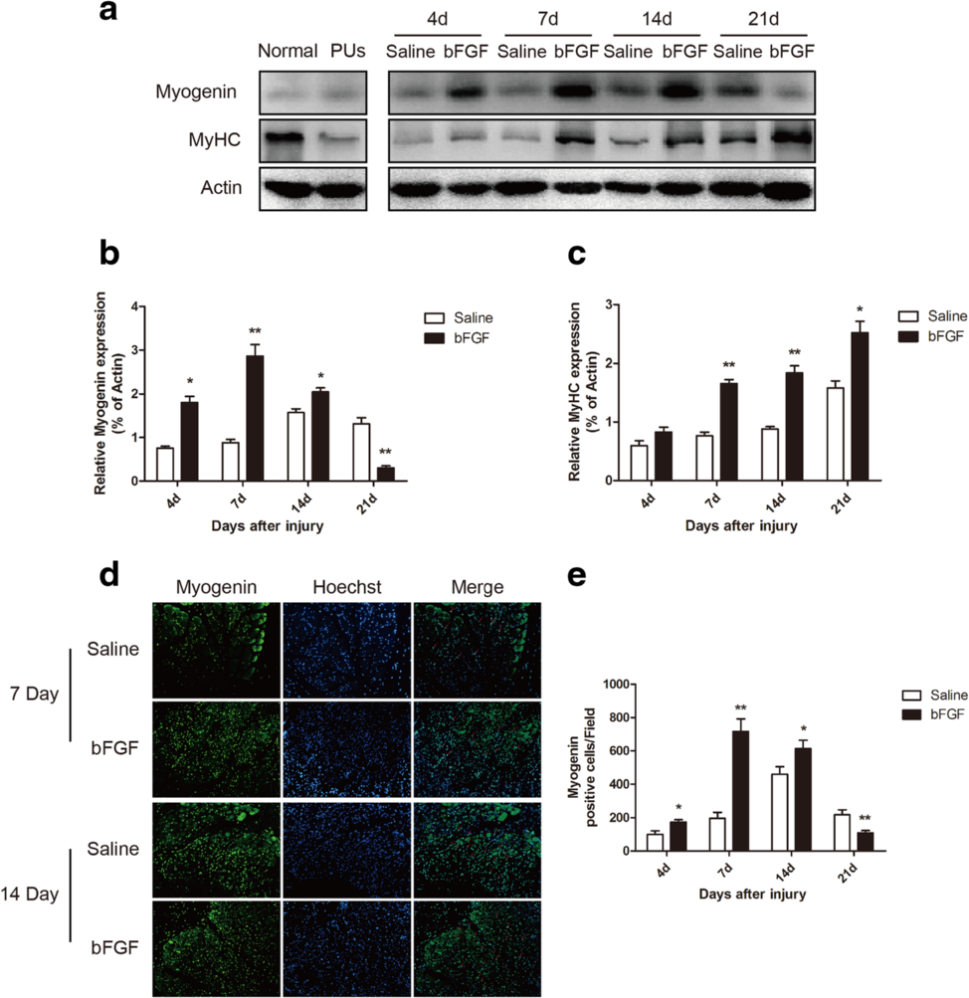
bFGF increased expression of myogenic markers myogenin and MyHC. **a** Western blot analysis of myogenin and MyHC protein expression in skeletal muscle tissues of saline group and bFGF group at indicated time. Actin was used as the loading control and for band density normalization. All of the experiments were repeated three times. **b**, **c** The optical density analysis of myogenin and MyHC protein. Data are expressed as Mean ± SEM (*n* = 3). **d** Photographs showing myogenin immunofluorescence (*green*, *red arrow* in merge pictures) on the 7th and 14th days in two groups (×200). **e** Graph of myogenin immunofluorescence in two groups. Data are expressed as mean ± SEM (*n* = 6). *P < 0.05 compared with saline group

Protein levels of the myogenic markers myogenin and MyHC were detected by western blot. Levels of myogenin were markedly up-regulated in the bFGF group compared to those in the saline group. Specifically, bFGF treatment resulted in myogenin levels that were induced 2.4-fold (*P* < 0.05) on the 4th day, peaked on the 7th day (3.2-fold, *P* < 0.05), were induced 1.3-fold (*P* < 0.05) on the 14th day, and were significantly decreased (0.2-fold, *P* < 0.05) on the 21st day (Fig. 3a, b). The protein levels of MyHC were also notably increased in bFGF group compared to the saline group. The protein levels of MyHC were induced 1.4-fold on the 4th day (*P* > 0.05), peaked on the 7th day (2.2-fold, *P* < 0.05) and 14th day (2.1-fold, *P* < 0.05), and were induced 1.6-fold (*P* < 0.05) on the 21st day (Fig. 3a, c). Immunofluorescence staining showed that myogenin was distributed in the nuclei of the skeletal muscle cells. Consistent with results of western blot, immunofluorescence staining showed that bFGF administration, compared to saline administration, increased the number of myogenin-positive cell 1.7-fold (*P* > 0.05), 3.6-fold (*P* < 0.05), 1.3-fold (*P* < 0.05), and 0.5-fold (*P* < 0.05) on the 4th, 7th, 14th, and 21st days after injury, respectively (Fig. 3d, e).

### bFGF up-regulated the phosphorylation of Akt and mTOR

**Fig. 4:**
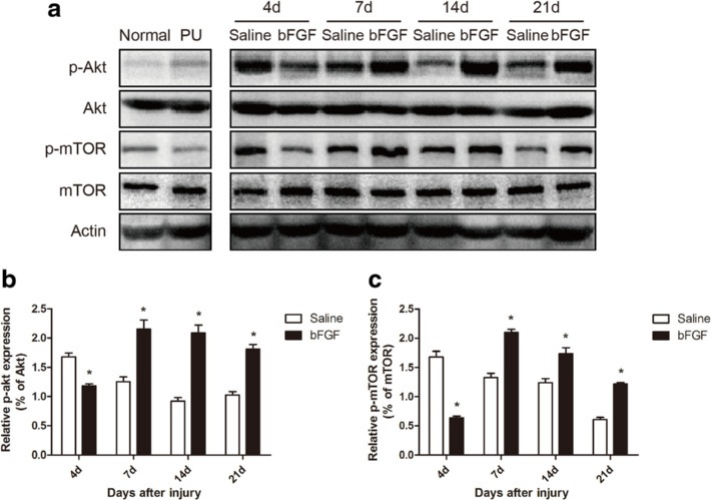
bFGF up-regulated the phosphorylation of Akt and mTOR. **a** Western blot analysis of phosphorylation of Akt and mTOR levels in skeletal muscle tissues of saline group and bFGF group at indicated time. Actin was used as the loading control, and total Akt and mTOR were used for band density normalization. All of the experiments were repeated three times. **b**, **c** The optical density analysis of phosphorylation of Akt and mTOR levels. Data are expressed as mean ± SEM (*n* = 3). *P < 0.05 compared with saline group

Activation of the PI3K/Akt/mTOR signaling pathway protects the heart against ischemia/reperfusion injury [19]. To investigate whether PI3K-Akt is involved in muscular regeneration, levels of phosphorylated Akt, and mTOR, which are the active forms of the proteins, were determined by western blot analysis. Levels of p-Akt were significantly decreased (0.7-fold, *P* < 0.05) after treatment with bFGF compared to saline group on the 4th day after injury. In contrast, on the 7th, 14th, and 21st days after injury, p-Akt levels were induced 1.7-fold (*P* < 0.05), 2.3-fold (*P* < 0.05), and 1.8-fold (*P* < 0.05) in the bFGF group compared to the saline group (Fig. 4a, b). A similar temporal expression pattern was found for p-mTOR. Specifically, p-mTOR levels were induced 0.4-fold (*P* < 0.05), 1.6-fold (*P* < 0.05), 1.4-fold (*P* < 0.05), and 2.0-fold (*P* < 0.05) in bFGF group on the 4th, 7th, 14th, and 21st days after injury, respectively (Fig. 4a, c). The up-regulation of the activities of these proteins suggest that the PI3K/Akt/mTOR signaling pathway is involved in the protection of bFGF in skeletal muscle regeneration after DTI.

## Discussion

PU is a serious and challenging health problem due to its widely variable and complex pathophysiology. The development of DTI after injury is multifactorial and mechanistically complex. The results in this study show that bFGF protected against compression-induced pathohistological damage in skeletal muscle through the promotion of cell proliferation, neovascularization, and up-regulation of MyHC and myogenin protein levels. We also suggest that activation of PI3K/Akt/mTOR signaling pathway may be involved in the regenerative effects of bFGF on injured skeletal muscle.

The major repair processes in response to muscle injury consist of a destruction phase, a repair phase, and a remodeling phase [9]. The destruction phase is characterized by the formation of a hematoma, necrosis of myofibers, degeneration, and inflammatory cell infiltration [20]. In our study, compressed muscle injury (in the saline group) leads to histopathological characteristics consistent with those of the destruction phase, specifically loss of muscle fibers, decreased fiber size, cellular swelling, inflammation infiltration, multiple focal necrosis, massive nuclei aggregation in interstitial space, and excess collagen deposition. These results are consistent with findings reported by Siu et al. [21]. Since the presence of degenerative characteristics was demonstrated in the underlying muscle tissue, this experimental model resembled pressure-induced DTI in a clinical situation [1, 22]. Excessive development of fibrosis hinders muscle regeneration and prevents full recovery, and reduction of fibrosis improves muscle regeneration in injured skeletal muscle [23]. In the current study, administration of bFGF accelerated wound healing and decreased wound areas. These changes were associated with increased formation of new myofibers and decreased fibrosis, ultimately improving recovery of skeletal muscle tissue.

Cell proliferation plays an essential role in the repair and regeneration of a number of types of damaged tissues. Expression of PCNA is also induced after skeletal injury. Song et al. demonstrated that PCNA significantly increased after burn and was associated with increased gene and protein expression of the myogenesis markers Pax7 and myogenin [24]. Moreover, muscle injury induced by intramuscular injection of bupivacaine hydrochloride in the soleus also significantly up-regulated [25], suggesting that up-regulation of cell proliferation also facilitates injured muscle regeneration. In our study, though PCNA-positive cells were found both in normal group and PU group, the number of PCNA-positive cells was higher in those with PUs. In addition, the number of PCNA-positive cells was significantly increased after bFGF treatment.

Impairment of the microcirculation after ischemic/reperfusion injury is a critical component in the pathogenesis of DTI. Vascular ingrowth into the muscle injury site is necessary to provide an adequate supply of oxygen and nutrients to the injured tissue and is correlated with the speed and quality of tissue repair. The effects of bFGF on neovascularization, along with other positive effects on tissue repair, have been investigated by the administration of bFGF on ischemia/reperfusion animal models [26]. Lee et al. demonstrated that local bFGF infusion into an ischemic limb not only promoted increased blood vessel density in the distal ischemic muscles but was also associated with the restoration of impaired muscle function [27]. Thus, it is reasonable to speculate that bFGF promotes angiogenesis, thereby increasing the blood supply to the injury site and facilitating injured muscle regeneration. Consistent with this idea, our data demonstrated that the number of CD31-positive cells and the capillary density at the injury site were significantly up-regulated after bFGF treatment.

Myogenin belongs to the family of myogenic regulatory factors (MRFs) which are composed of MyoD, myogenin, myf-5, and myf-6, and its expression is rapidly followed by terminal withdrawal from the cell cycle and expression of muscle-specific structural proteins [28]. MRFs play an important role in myoblast differentiation and in the formation of multinucleated myotubes, facilitating injured muscle regeneration [29, 30]. Mofarrahi et al. showed that Angiopoietin-1 increased expression of the MRFs (MyoD and Myogenin) and enhanced skeletal muscle regeneration in response to fiber injury [31]. The MyHC protein is critical for physiological muscle function [32], and like MRFs, serves as a myogenic marker in muscle development and regeneration. In our study, myogenin expression level peaked on the 7th, and 14th day after injury, and significantly decreased on the 21st day, suggesting that bFGF facilitated myogenesis, as indicated by higher MyHC expression levels. This temporal profile of myogenin expression implied that bFGF exerted its biological roles during the first 2 weeks after injury.

Muscle mass is controlled by complex cell signaling pathways that regulate muscle protein synthesis and degradation. The serine/threonine protein kinase Akt is an important mediator of phosphatidylinositol-3 kinase (PI3K) signaling in different tissues and regulates multiple aspects of cellular functions, including survival, growth, and metabolism [33]. The mammalian target of rapamycin (mTOR), a downstream component of the PI3K-Akt signaling pathway, plays a regulatory role in translation initiation, protein synthesis, and muscle hypertrophy [34]. Akt has been shown to induce transcription of muscle-specific genes, resulting in myoblast differentiation [35, 36]. Furthermore, the Akt/mTOR pathway is a crucial regulator of skeletal muscle hypertrophy and can prevent muscle atrophy [34]. Previous studies have reported that PI3K activity is necessary for the activation of the myogenic program [37]. Consistent with this, the PI3K-Akt-mTOR pathway is activated in muscles after acute contusion in mice [38]. bFGF-mediated activation of the PI3K-Akt pathway is a potent mediator of muscle differentiation [39] and protects the heart against ischemia/reperfusion injury [19, 40]. In our study, bFGF treatment significantly induced phosphorylation of Akt and mTOR, thereby activating them. The phosphorylation of Akt and mTOR was in turn associated with increased cell proliferation, differentiation, and myogenesis. However, on the 4th day after injury, phosphorylation of Akt and mTOR was notably decreased in bFGF-treated rats compared to those in the saline group. We speculated that this decrease in the bFGF-treated group may be related to macrophage phagocytosis, which leads to bFGF degradation. Therefore, the development of new drug delivery systems that can protect the protein against degradation should be the focus of future studies.

As we described above, satellite cells function as adult muscle stem cells and are responsible for regenerating muscle. Previous studies have demonstrated that bFGF mRNA is expressed by skeletal satellite cells [41] and that bFGF enhances satellite cell proliferation [42]. Another study reported that fibroblast growth factor receptors (FGFR1 and FGFR4) were expressed at relatively high levels in quiescent satellite cells, and ablation of FGFR1 impaired bFGF-mediated proliferation of satellite cells at the myofibers niche but did not abolish the capacity for muscle regeneration [43], suggesting a more complex relationship between FGF-mediated satellite cell proliferation and regeneration. However, in our study, we did not explore the intricacies of the mechanisms underlying bFGF-mediated satellite cell proliferation and regeneration in the DTI model, so further study is warranted. The results of our study showed that bFGF activated PI3K-Akt-mTOR signaling pathway, but it remains unclear that whether this pathway is an essential pathway improving recovery of injured skeletal muscle, though Akt/mTOR pathway is a crucial regulator of skeletal muscle hypertrophy and prevents muscle atrophy [34]. Further studies using knock-out mice should explore the role of the PI3K-Akt-mTOR signaling in the recovery of injured skeletal muscle. Another defect in our study is whether ERK1/2 is involved in bFGF-induced injured skeletal muscle recovery. As we have known, ERK1/2 signaling is one of downstream pathways of FGF family protein [44], and activation of ERK1/2 is also related to injured muscle regeneration [45]. Therefore, whether ERK1/2 signaling pathway is involved in the bFGF-promoted injured muscle recovery needs further study.

## Conclusions

Our results showed that DTI induced skeletal muscle degradation and that bFGF improved injured skeletal muscle recovery. We propose that bFGF improves skeletal muscle recovery by promoting cell proliferation and angiogenesis and facilitating myogenesis via activating the PI3K-Akt-mTOR signaling pathway. Our findings suggest that bFGF can be developed as a potential therapeutic candidate for the treatment of PUs, especially DTI, in clinical trials.

## Abbreviations

bFGF, basic fibroblast growth factor; DTI, deep tissue injury; FGFRs, fibroblast growth factor receptors; ERK1/2, extracellular signal–regulated kinases; FGFs, fibroblast growth factors; HE, hematoxylin–eosin; MRFs, myogenic regulatory factors; mTOR, mammalian target of rapamycin; MyHC, myosin heavy chain; PCNA, proliferating cell nuclear antigen; PI3K, phosphatidylinositol-3-kinase; PUs, pressure ulcers

## Additional file

Additional file 1: Supporting information. (PDF 440 kb)
