# Supplementary material for: Myoprotective effects of bFGF on skeletal muscle injury in pressure-related deep tissue injury in rats
Source: Burns Trauma. 2016 Aug 17;4:26. doi: 10.1186/s41038-016-0051-y (PMC4987989; doi:10.1186/s41038-016-0051-y)

Supporting information

DTI Model

Male adult Sprague Dawley (SD) rats (age 12 weeks) were individually housed in plastic cages with stainless-steel covers to prevent the accidental dislocation of the magnets and to prevent tampering with the resultant ulcer by other animals. The limb hair was shaved. A template was used to mark the location of the magnetic plates to assure a consistent placement on each animal. The limb was placed between 2 round ceramic magnetic plates that had 8 mm diameter and were 5.0 mm thickness, with an average weight of 2.0 g and 4000 G magnetic force (**Fig. S1A**). The resultant "pinch" procedure was designed to leave a 5.0-mm skin-muscle bridge between the two magnets (**Fig. S1B**). This process created 400 mmHg compressive pressure between the two plates that has been documented to be necessary to cause local tissue ischemia by Fang Lin and coworkers (1).

Three ischemia/reperfusion cycles were used in each animal to initiate decubitus ulcer formation. A single ischemia/reperfusion cycle consisted of a 12 hr period of magnet placement followed by a release period of 12 hr. Animals were not immobilized, anesthetized, or otherwise treated during the ischemia/reperfusion cycles. Animals were housed under a 12 hr light/dark cycle in a pathogen-free area with free access to water and food. All efforts were made to minimize the number of animals used and their suffering. Based on our observations, the animals resumed normal activity within a few minutes of magnet placement. The animals were able to tolerate the additional weight of the magnets without any difficulty.

Reference

1. Lin F, Pandya A, Cichowski A, et al. J Tissue Viability 2010: 19: 67-76.

Figure S1. DTI PU model and experiment design. (A, B) Demonstration of the application of compression load to bilateral shanks of a rat. **(C)** Schematic diagram of whole experiment design.

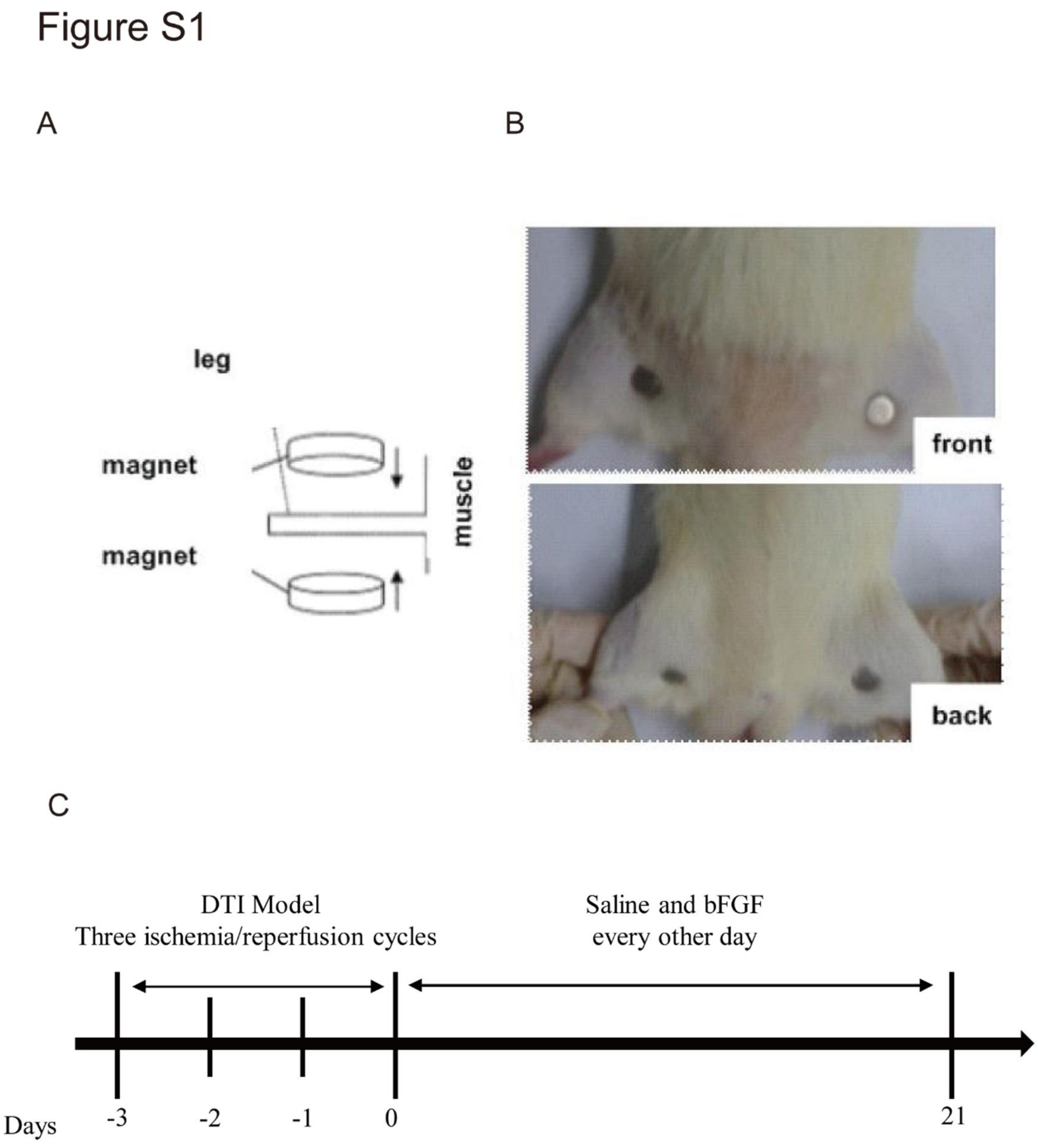

Figure S2. Administration of bFGF decreased wound area. **(A)** Representative images showing wound areas in rats treated with saline, and bFGF (10 μg /0.1ml) at different time-points following DTI. **(B)** Graph showing the wound areas in the saline group and bFGF group. Data are expressed as Mean ± SEM (n = 8). *P < 0.05 compared with saline group at the indicated time.

Figure S2

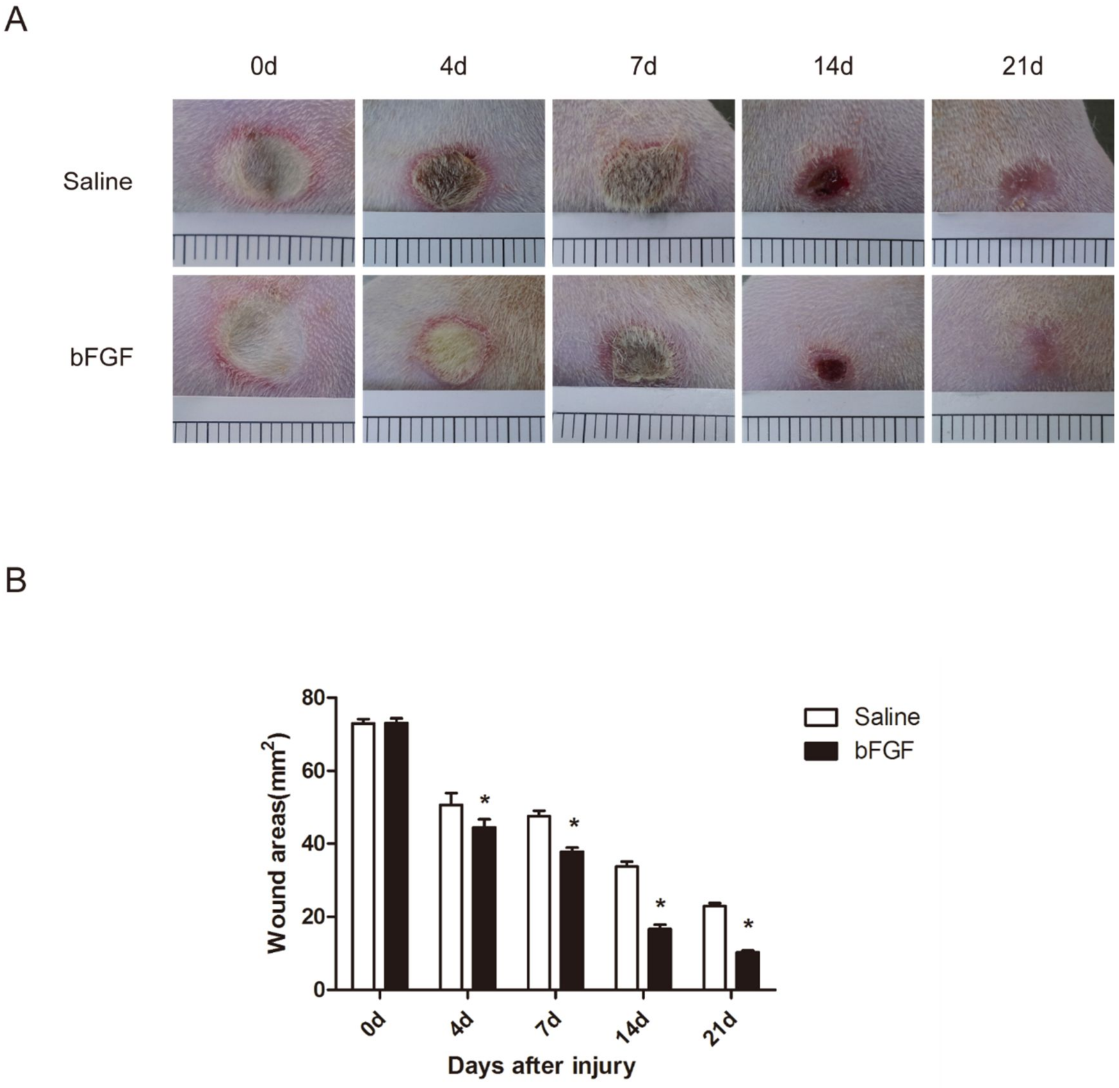

Supplement: Additional file 1: — Supporting information. (PDF 440 kb) [file 41038_2016_51_MOESM1_ESM.pdf]
